# The Recognition of and Reactions to Nucleic Acid Nanoparticles by Human Immune Cells

**DOI:** 10.3390/molecules26144231

**Published:** 2021-07-12

**Authors:** Dominika Bila, Yasmine Radwan, Marina A. Dobrovolskaia, Martin Panigaj, Kirill A. Afonin

**Affiliations:** 1 Faculty of Science, Institute of Biology and Ecology, Pavol Jozef Safarik University in Kosice, 04154 Kosice, Slovakia; dominika.bila@student.upjs.sk; 2 Nanoscale Science Program, Department of Chemistry, University of North Carolina at Charlotte, Charlotte, NC 28223, USA; yradwan@uncc.edu; 3 Nanotechnology Characterization Laboratory, Cancer Research Technology Program, Frederick National Laboratory for Cancer Research Sponsored by the National Cancer Institute, Frederick, MD 21702, USA; marina@mail.nih.gov

**Keywords:** nucleic acid nanoparticles (NANPs), immunorecognition, immunoreaction, Toll-like receptors, cytokine storm syndrome, complement activation-related pseudoallergy

## Abstract

The relatively straightforward methods of designing and assembling various functional nucleic acids into nanoparticles offer advantages for applications in diverse diagnostic and therapeutic approaches. However, due to the novelty of this approach, nucleic acid nanoparticles (NANPs) are not yet used in the clinic. The immune recognition of NANPs is among the areas of preclinical investigation aimed at enabling the translation of these novel materials into clinical settings. NANPs’ interactions with the complement system, coagulation systems, and immune cells are essential components of their preclinical safety portfolio. It has been established that NANPs’ physicochemical properties—composition, shape, and size—determine their interactions with immune cells (primarily blood plasmacytoid dendritic cells and monocytes), enable recognition by pattern recognition receptors (PRRs) such as Toll-like receptors (TLRs) and RIG-I-like receptors (RLRs), and mediate the subsequent cytokine response. However, unlike traditional therapeutic nucleic acids (e.g., CpG oligonucleotides), NANPs do not trigger a cytokine response unless they are delivered into the cells using a carrier. Recently, it was discovered that the type of carrier provides an additional tool for regulating both the spectrum and the magnitude of the cytokine response to NANPs. Herein, we review the current knowledge of NANPs’ interactions with various components of the immune system to emphasize the unique properties of these nanomaterials and highlight opportunities for their use in vaccines and immunotherapy.

## 1. Introduction

### Nucleic Acid Nanoparticles

Nanomedicine is an application of nanotechnology in medical settings for diagnosis, treatment, and prevention. It exploits unique chemical, physical, and biological properties of materials at the nanoscale. One of the perspective branches of nanomedicine is nucleic acid nanotechnology, which uses nucleic acids—DNA, RNA, and their various modifications—to design and formulate nanostructures for therapeutic applications [1].

Due to the programmability and the intrinsic functions of nucleic acids, single-stranded DNA or RNA molecules are rationally designed into modular nucleic acid nanoparticles (NANPs) that are easily customized into supramolecular three-dimensional structures exclusively made of nucleic acids. RNA and DNA form canonical and non-canonical base pairings to assemble into various higher-order structures that serve as a basis for the assembly of different nanostructures including rings, fibers, and polygons [1,2,3,4,5,6]. Advantageously, the choice of nucleic acid components provides tunability for the physicochemical properties, biological activities, and multifunctionality of NANPs. Many experiments in biotechnology and biomedicine propose applications of NANPs as carriers of bioactive compounds, molecular tools for imaging and biosensing, scaffolds for biochemical reactions, or multifunctional nanoparticles combining the previous functions into one complex [1,7,8,9]. The rapidly evolving field of nucleic acid nanotechnology had provided multiple synthesis methods for NANPs, established their characterization techniques in vitro and in vivo, and produced proof-of-concept data for using NANPs in various therapeutic applications [10,11,12,13].

NANPs can achieve biomedical functions by delivering therapeutic nucleic acids (TNAs) that are designed to perform key functions in gene regulation and expression and protein synthesis to serve in therapeutic applications. The modular functionalization of NANPs with aptamers, antibodies, or small molecules for their targeted delivery allows NANPs to integrate and deliver various TNAs into cells for synergistic therapeutic effects. However, despite these developments, NANPs have yet to advance to clinical translation due to concerns that need to be investigated and resolved including their specific delivery to target cells, their enzymatic degradation, and their ability to induce an immune response upon cellular uptake [4,12,14,15]. While targeting and stability are not immediate life-threatening issues, the excessive immune recognition of NANPs and overreaction by immune cells can have potentially deleterious effects. Thereby, the immunostimulatory properties of NANPs are being extensively investigated [14,16,17].

Several physicochemical properties of NANPs determine their recognition by the immune cells; the most notable properties are 3D structure, composition (RNA to DNA ratio), molecular size, and the NANP’s sequence. In addition, the immune response could be modulated by the type of delivery carriers used [6,14,18,19,20,21,22]. The proper design of NANPs with respect to immunostimulatory properties has the potential to activate innate and adaptive immune responses by activating nucleic acid immune sensors, thus having high potential as vaccine adjuvants and pan-antivirals [2,14,20,21,23,24,25]. Our emerging knowledge about the individual immunostimulatory abilities of nucleic acids aids in the design of safe NANPs, but it must be stressed that because of the effects of structure, the immunological characteristics of NANPs are not the sum of their individual components. Therefore, each NANP assembly must be experimentally tested and safety validated.

## 2. Recognition and Reaction of Immune Cells to Nucleic Acids

Immune cells are equipped with an extensive portfolio of so-called pattern recognition receptors (PRRs) that detect pathogen-associated molecular patterns (PAMPs) and damage-associated molecular patterns (DAMPS). The first line of PRRs include Toll-like receptors (TLRs) located on cell membranes (TLR1, TLR2, TLR4, TLR5, TLR6, TLR10) and in the endosomal compartment (TLR3, TLR7, TLR8, and TLR9) followed by RIG-I-like receptors (RLRs) or DNA sensor cyclic GMP–AMP synthase (cGAS) situated in the cytosol [20,26].

**Figure 1:**
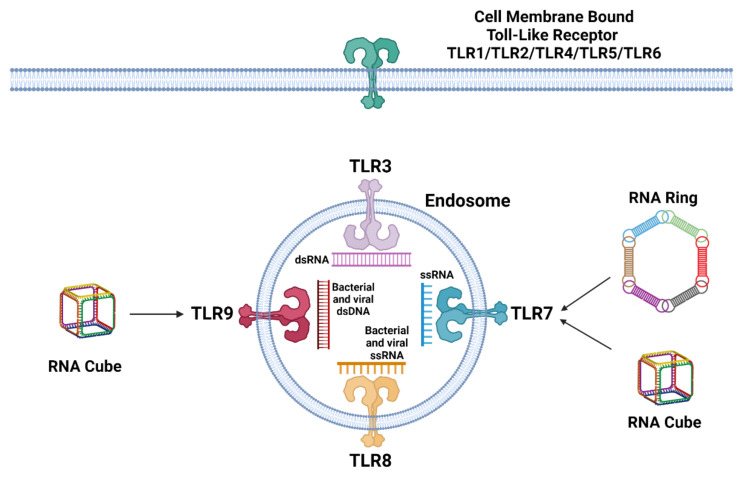
Toll-like receptors. Cell membrane-bound TLRs include TLR1, TLR2, TLR4, TLR5, and TLR6, while endosomal TLRs include TLR3, TLR7, TLR8, and TLR9. TLR3 recognizes double-stranded RNA (dsRNA). TLR8 recognizes bacterial and viral single-stranded RNA (ssRNA). TLR7 recognizes single-stranded RNA (ssRNA), as well as ring and cube RNA. TLR9 recognizes bacterial and viral double-stranded DNA (dsDNA), along with cube RNA. It is important to note that RNA cube triggers the activation of TLR9 and TLR7 only after its delivery inside the cell using a carrier such as L2K.

TLR sensing of nucleic acids is specific for RNA or DNA recognition and resides in the endosomal compartment, where TLR3 is specific for double-stranded RNA, including small interfering RNA (siRNA), TLR7 functions as a single-stranded RNA receptor, TLR8 is specific for bacterial and viral RNA immune recognition, and finally, TLR9 responds to bacterial and viral DNA (Figure 1) [20,26]. Recognition of nucleic acids from non-cellular origins activates a complex network of signaling cascades that usually culminates in the expression of interferons (IFNs), including other cytokines and various chemokines. The general goal of the response is to alarm adjacent cells and recruit cells of adaptive immunity. The recognition of nucleic acids by TLRs causes signal transduction through Toll/interleukin-1 receptor (TIR)-containing signaling adaptors, TRIF, or MyD88 [27,28]. The downstream acceptor of these signals is NF-κB which, upon activation, translocates into the nucleus and induces the expression of pro-inflammatory genes [26,29]. NF-κB is functioning in both innate and adaptive immune cells. In addition to the mediation of macrophage inflammatory responses, NF-κB promotes the activation and differentiation of T cells and the maturation and differentiation of B cells [30,31]. Finally, the expression of IFNs modulates further immune defense via paracrine and autocrine signaling through the transcription of IFN-stimulated genes (ISGs). The main effector functions of ISGs are to target pathways and functions required during the pathogens’ life cycle as well as to enhance innate immune signaling. In addition, ISGs encode proapoptotic proteins that lead cells to apoptosis under specific conditions [32,33].

Intracellular surveillance of RNA is carried out by RLRs, mainly the retinoic acid-inducible gene-I protein (RIG-I) and melanoma differentiation-associated protein 5 (MDA5) located in the cytosol, although the presence of RIG-I has also been observed in the nucleus. RIG-I and MDA5 are activated by binding short double-stranded RNA (dsRNA) with a 5′-triphosphate and 5′-diphosphate or long dsRNA structures, respectively. Furthermore, for the most efficient activation of RIG-I, the blunt end is required as well as a short double-stranded sequence. Activated RIG-I interacts with the mitochondrial antiviral signaling protein (MAVS) residing on the mitochondrial membrane or peroxisomes. Finally, kinase complexes activated by MAVS induce transcription through IRF3, IRF7, and NF-κB. The main cytoplasmic sensors of dsDNA are cyclic GMP-AMP synthase (cGAS) and IFNγ-inducible protein 16 (IFI16), which is also located in the nucleus, where it probably detects naked viral DNA. After binding dsDNA, cGAS synthesizes the second messenger 2′3′-cyclic-GMP-AMP (cGAMP) that subsequently mobilizes the stimulator of IFN genes (STING) on the endoplasmic reticulum that again induces the transcription of antiviral genes through IRF3 and NF-κB [34,35,36].

## 3. Recognition and Reaction of Immune Cells to NANPs

NANPs demonstrate different interactions with various types of immune cells, that, unlike traditional nucleic acid therapeutics, are also determined by the type of carrier or complexation agent used for NANPs’ intracellular delivery. Without such agents, plain NANPs are invisible to the immune cells and do not trigger cellular immunological responses. For example, flow cytometric analysis of freshly collected human peripheral blood mononuclear cells (PBMCs) treated with a carefully chosen panel of NANPs with various compositions (RNA, DNA) and connectivity (globular, planar, and fibrous) revealed that after complexation with Lipofectamine 2000 (L2K), most NANPs are associated with the monocyte fraction and less with lymphocytes. Subsequent confocal microscopy showed that in monocytes, L2K-complexed NANPs were located inside the cells. Using a dye labeling the endolysosomal compartment and an inhibitor of endosomal uptake, it was observed that unlike lymphocytes, monocytes transport L2K-complexed NANPs into their interiors via endosomes. Overall, phagocytosis and endosomal acidification are key processes for L2K-complexed NANPs’ uptake by monocytes. A further functional study indicated that scavenger receptors (SRs) are the most probable receptors involved with binding and internalization of L2K-complexed NANPs. In addition, the inhibition of SRs also prevented the expression of IFN-α in response to L2K-complexed NANPs [21]. Scavenger receptors are a heterogenous group of cell surface receptors that recognize a broad range of ligands; therefore, we currently do not know the mechanism of how SRs recognize NANPs [37]. Without L2K, NANPs did not show any signs of internalization by immune cells present in PBMCs and did not trigger the activation of PRRs or interferon responses.

Plasmacytoid dendritic cells (pDCs) play a key role in linking the innate immune and adaptive response, and although they constitute less than 1% of the monocyte fraction, pDCs, in comparison with isolated monocytes and myeloid DCs, respond to L2K-complexed NANPs with the strongest expression of type I and III IFNs. While in all fractions, RNA cubes appear as the strongest inducer of IFN response, pDCs activated IFNs regardless of the composition (DNA vs. RNA) or 3D structure. The depletion of pDCs from PBMCs leads to a dramatic reduction of IFN production, which means that pDCs are the primary source of immune reaction to NANPs. Interestingly, the distinct expression profile of IFN-α, IFN-β, IFN-ω, and IFN-λ between whole PBMCs and isolated pDCs implies that most likely, there is cellular crosstalk among PBMC subpopulations, which determines the overall response to NANPs [21].

The next important question is, which PRRs are responsible for the recognition and triggering of signaling cascades? The application of a pan oligonucleotide inhibitor of endosomal TLR signaling completely prevented the induction of IFN response upon treatment of PBMCs with any L2K-complexed NANPs used in the study. Similar results were observed in purified pDCs. The model HEK293 cell lines overexpressing either TLR3, TLR7, TLR8, or TLR9 were used to rule out which TLR type recognizes respective NANPs. In this model, the globular NANPs (RNA cubes) were sensed by TLR7, and RNA fibers were sensed by the rest of the examined TLRs [21].

In a follow-up study, we downregulated TLR7 and TLR9 expression in PBMCs by a mix of siRNAs. TLR7 and TLR9 were chosen as TLRs expressed in pDCs that are the primary IFN producers in the PBMC pool. However, the interpretation of observed data is complicated by different levels of downregulation of TLRs among the cells isolated from different healthy donors. Even the extent of silencing between TLR7 and TLR9 in one donor varied. The possible explanation may lay in the inter-individual sequence heterogeneity or regulation of TLRs’ expression. The significant reduction in IFN response for the L2K-complexed RNA cubes was observed in two out of three donors with silenced TLR7, while no decrease in IFN production was detected upon treatment with L2K-complexed RNA fibers or DNA cubes. The downregulation of TLR9 prevented IFN response only in culture from one donor treated with RNA cubes and from another donor treated with RNA rings [20]. Taken together, TLR7 is responsible for RNA rings’ and cubes’ immune recognition but not DNA cubes nor RNA fibers (Figure 1).

## 4. What Makes NANPs Immunostimulatory?

The recognition of NANPs by the cell defense system depends on several physicochemical characteristics, including composition, 3D structure, sequence, shape, size, and connectivity. One of the first observations that the composition of NANPs (number of RNA vs. DNA strands that enter the composition of a particular NANP) affects their immune recognition came from the earlier study of functionally interdependent shape-switching nanoparticles where we noted that all examined NANPs triggered an IFN-α response, but NANPs assembled from six RNA strands were the most immunostimulatory [38]. A similar trend was observed in a study implementing a new RNA tetra-U helix linking motif in triangles with different DNA vs. RNA composition. In a model of human microglia-like cells, the transfection of RNA triangles induced the highest level of IFN-β production, followed by hybrid DNA/RNA triangles. No expression of IFN-β was stimulated by DNA triangles [19].

**Figure 2:**
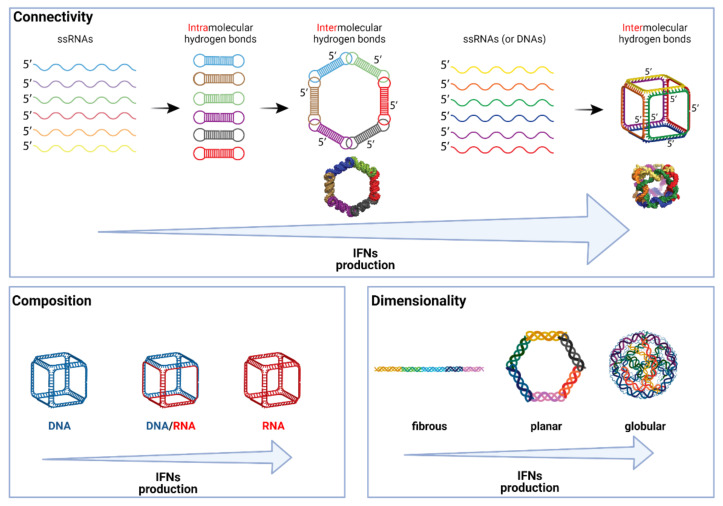
Influence of physicochemical properties on immune stimulation. The main characteristics of NANPs that affect their immunostimulation are connectivity (how individual NANP strands are assembled), composition (number of RNA strands vs. DNA), and dimensionality (3D shape).

Several structure–activity relationship models that link the physicochemical properties of NANPs to their immunostimulation have emerged from a larger analysis of 25 different NANPs [21]. First, globular RNA cubes proved to be the most immunostimulatory NANPs. In comparison to DNA cubes that have almost identical shape and size, RNA cubes induced not only IFN-α and IFN-ω as DNA NANPs did, but also IFN-β and type III IFNs (IFN-λ). In addition, RNA cubes were more immunostimulatory than any other RNA-based NANPs (planar rings or fibers), and planar DNA or RNA structures were more immunostimulatory than chemically corresponding fibrous nanoobjects (Figure 2). In all these examples, NANPs were delivered to the cells using L2K.

The chemical complexity or diversity of assembled NANPs can be increased by the incorporation of modified bases in individual strands. Especially for RNA bases, the diverse modifications play significant roles in RNA stability and affect the immunostimulatory potential [39]. Various experiments have described that the modification of RNA (herein siRNA) helps to circumvent TLR signaling and renders modified RNA immunoquiescent [40]. Therefore, it is interesting that when used with a carrier (L2K or DOTAP), triangular NANPs that consisted of a DNA strand in their center and 2′fluoropyrimidine-modified RNA strands on their sides induced IFN-β and IL-6 production, unlike all DNA NANPs and NANPs composed of a DNA center and unmodified RNA sides. The results suggest that the presence of 2′fluoro-modification significantly enhances the immunoreactivity of DNA-containing NANPs. The NANPs with RNA in the center and 2′fluoropyrimidine-modified RNA sides stimulated IFN-β and IL-6 production similarly to all RNA NANPs and NANPs composed of an RNA center and DNA sides. This indicates that 2′fluoropyrmidine modification does not affect the immune mediator response. The fully 2′fluoropyrimide-modified RNA triangles stimulated significant IFN-β and IL-6 production similarly to NANPs with either an RNA center and 2′fluoropyrimidine-modified RNA sides or NANPs consisting of a DNA center and 2′fluoropyrimidine-modified RNA sides [13]. Surprisingly, the incorporation of 2′fluoro-modifications into RNA NANPs abrogated the activation of TLR7 in the HEK293 reporter cell line but failed to avoid RIG-I dependent immune responses [14].

The ability to design complementary NANPs (also called anti-NANPs) that are assembled from the reverse complementary strands of evaluated NANPs allows for examining the effects of the sequence of NANPs on the ability to activate an IFN response. NANPs and anti-NANPs had completely different sequences but nearly identical 3D shapes. The RNA rings and DNA cubes were able to stimulate similar levels of IFN to their anti-NANPs analogs and anti-RNA cubes maintained the high response, which indicates that the NANPs’ sequences are less important for immunostimulation than their 3D shape and composition (RNA vs. DNA). Except for the RNA rings and RNA fibers that are assembled from pre-formed monomers, all other studied NANPs (cubes, polygons, tetrahedrons, and DNA fibers) create intermolecular bonds (Figure 2). Indeed, free-unpaired nucleotides (ssUs) have enhancing effects on immunogenicity, but it appears only for globular NANPs such as RNA cubes. Interestingly, PBMCs from donors that demonstrated a higher IFN response to a TLR agonist (ODN 2216) reacted stronger to RNA cubes with nine ssUs in their corners than to cubes with a lower number of ssUS (three and six). On the other side, blood cells with lower reactions to the administered TLR agonist induced a similar IFN expression irrespective of the numbers of ssUs.

The size of the nanoparticles is one the main characteristics with potential impact on interactions with cells. Similar to the case of the number of free nucleotides in RNA cubes, the difference was observed only in donor cells with high reactions to ODN 2216, where hexagons activated the stronger response than three-, four-, or five-sided RNA polygons. Adjusting the mass of smaller polygons to be equal to or larger than that of the larger polygons had no effect on IFN production. In cells with low activation by ODN 2216, there was no observed difference between individual NANPs. In the case of DNA polygons, no significant differences were detected between different sizes of NANPs [21].

## 5. Delivery Method/Carrier: An Unexpected Immunomodulator

**Figure 3:**
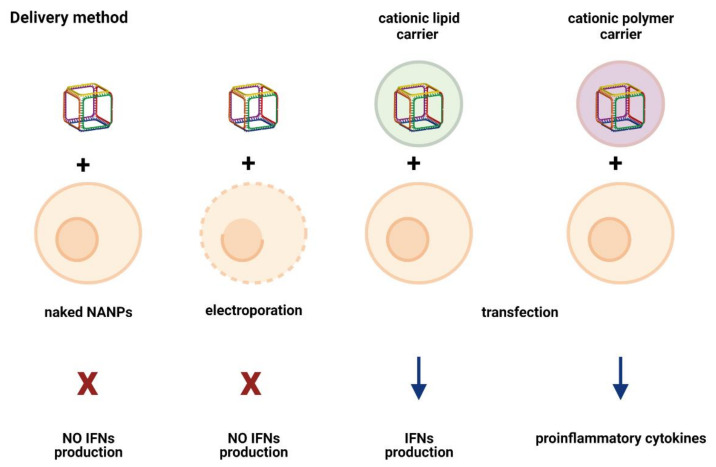
Delivery method and type of carrier affects cellular immune response. Naked NANPs do not trigger IFN response even upon transport to the cytosol via electroporation. Cellular defense is activated by NANPs only if they are in complex with carrier. The type of delivery carrier determines the spectrum of cytokines produced in response to NANPs.

The immunostimulatory potential of NANPs is significantly influenced by the employed delivery method. The NANPs without a delivery agent are not efficiently internalized and thus do not induce IFN production. Even if naked NANPs are delivered to cells via electroporation, no production of IFNs was detected in response to any of the tested NANPs (Figure 3). Moreover, electroporated cells lose the ability to respond to other known inducers of IFN response, such as TLR9 agonist ODN 2216, although the addition of ODN2216 to the non-electroporated cells resulted in high levels of type I and III IFNs. The results suggest that electroporation negatively affects endosomal TLR signaling, thereby affecting the ability of cells to elicit an immune response [20].

The importance of complexing the NANPs with a carrier for immunorecognition was demonstrated in a study that tested the ability of RNA cubes to induce the type I IFN immune response. The NANPs added to the cell cultures without a delivery carrier were incapable of stimulating an IFN response, while the NANPs complexed with L2K showed the ability to induce the secretion of both type I and type III IFNs. On the other side, ODN 2216, which was used as a positive control, stimulated an IFN response regardless of its complexation with L2K. The application of carrier itself did not cause the induction of the IFN response [21]. L2K does not affect NANPs’ structures. Not surprisingly, different carriers demonstrate distinct transfection efficiencies for the same NANP [14].

Although the delivery of NANPs remains a challenge, new carriers are constantly introduced and tested. For instance, the immunostimulatory ability of the lipid-based carrier versus a cationic amphiphilic copolymer was compared. The NANPs delivered via the lipid-based carrier stimulated the production of both IL-6 and IFN-β. In contrast, when the NANPs were delivered using an amphiphilic copolymer, no statistically significant presence of IL-6 or IFN-β was detected. The results suggest that the employment of a cationic amphiphilic copolymer as a delivery carrier can reduce the immunostimulation, therein decreasing off-target effects [41].

Another recent study compared a lipid-based carrier (L2K) and dendrimers (PAMAM) to determine whether the spectrum and the magnitude of the cytokine response to RNA and DNA cubes depend on the type of the utilized carrier. The results showed significant differences in the induction of type I and type III IFNs and pro-inflammatory cytokines between NANPs delivered utilizing a lipid-based carrier and those delivered via dendrimers. The NANPs complexed with L2K stimulated type I and type III IFNs, while the complexation of NANPs with dendrimers did not induce an IFN response. A remarkable difference was observed for cytokines associated with stress and danger (TNFα, IL-1 β, IL-6). The NANPs delivered via L2K did not stimulate a danger response, whereas those complexed with dendrimer induced the production of the stress- and danger-associated pro-inflammatory cytokines. The examination of chemokines (IL-8, MIP-1α, MIP-1β, MCP-1, MCP-2, and RANTES) showed that dendrimers alone did not stimulate any of the chosen chemokines, while the L2K carrier alone induced the production of all examined chemokines but MCP-2. The induction of MCP-2 was detected only when NANPs were complexed with the lipid-based carrier but not for dendrimer-complexed NANPs. Intriguingly, the induction of IL-8, MIP-1α, MCP-1, and RANTES was comparable between NANPs complexed with the lipid-based carrier and complexed with dendrimers. These results support the hypothesis that the type of carrier used for NANPs’ delivery significantly alters their ability to stimulate the immune response, both quantitatively and qualitatively [4].

## 6. Complement Activation-Related Pseudoallergy (CARPA) and Cytokine Release Syndrome (CRS)

The systemic administration of pharmacologic or biologic agents can cause a strong and serious response in immune cells. Infusion-related reactions (IRs), a form of anaphylaxis or other hypersensitivity reactions occurring within minutes to hours of infusion, are immune-mediated adverse effects that occur after the administration of various products, including low-molecular-weight drugs, antibodies, and recombinant proteins, therapeutic nucleic acids, and nanotechnology-formulated products. Frequently observed symptoms in patients with IRs comprise flushing or rash, chest and back pain, dyspnea, wheezing, chills, or fever. These manifestations can lead to serious and potentially fatal consequences. Therefore, accurate assessments and early intervention are crucial when these symptoms occur. When IRs are triggered by the complement system, anaphylactoid reactions or CARPA occur. CARPA has the same symptoms and timeline of development as immediate type hypersensitivity (ITH) reactions. However, in contrast to the ITH, which are mediated by the antigen-specific IgE, CARPA is triggered by the complement. Both CARPA and CRS, also known as cytokine storm, are common, and the best understood mechanisms of IRs are associated with nanotechnology-formulated products [42].

The fundamental processes of CARPA include complement system activation, stimulation of blood cells and secretory cells, and the response of effector cells to mediator presence. The complement is activated via an initial trigger. The initial trigger can be radiocontrast agents, therapeutic antibodies, micellar and liposomal formulations, or nanoparticles. After the activation of the complement, anaphylatoxins are released. The anaphylatoxins are primary mediators that bind to target secretory cells (macrophages, mast cells, basophils, other phagocytic cells, and leukocytes), resulting in a release of secondary mediators that include cytokines, proteases, histamine, tryptase, prostaglandins, platelet-activating factor, thromboxane A2, and leukotrienes. The indications of CARPA are like those that occur with common allergies, with some unique exceptions. The most frequent symptoms are asthma, chest pain, chills, confusion, coughing, dermatitis, diaphoresis, dyspnea, edema, erythema, fever, headache, hypertension, hypotension, hypoxemia, nausea, rash, and wheezing [43]. The significant distinguishing feature is that the reaction arises after the first exposure to the drug and then decreases upon repeated exposure. In the case of NANPs, the lipid-based carrier is the most common cause of complement activation, which can subsequently lead to CARPA [44]. The large size and positive or negative surface charge of liposomes were shown to promote complement activation, whereas liposomes of a smaller size and neutral charge had reduced ability for activation [45]. In addition, the susceptibility of liposomes for complement activation was demonstrated to depend on dose and, in the case of PEGylated liposomes, on the presence of anti-PEG antibodies.

**Figure 4:**
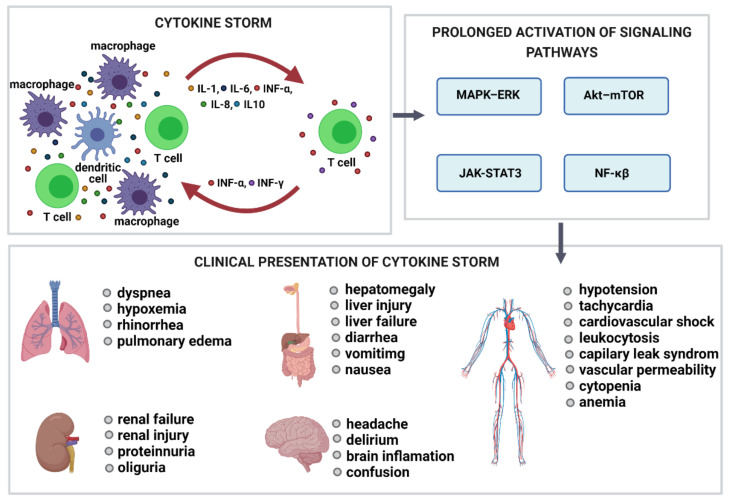
Cytokine storm. Cytokine storm is the result of the rapid release of numerous pro-inflammatory cytokines, including INF-γ, INF-α, IL-1, and IL-6. T cells, macrophages, neutrophils, and NK cells are most often involved in the cytokine storm pathogenesis. The activation of primary T cells or immune cells’ lysis stimulates the production of IFN-γ and TNF-α, which activate other immune cells and endothelial cells to release more pro-inflammatory cytokines. The excessive production of IL-6 constantly activates the JAK–STAT3, Akt–mTOR, and MAPK–ERK signaling pathways. Their prolonged activation stimulates immune cells to produce more cytokines, which causes hyperinflammation and multiple organ failure. JAK–STAT3, Janus kinase-signal transducer and activator of transcription 3; MAPK, mitogen-activated protein kinase; mTOR, mammalian target of rapamycin; NF-κB, nuclear factor κB.

The CRS is a systemic inflammatory response caused by the excessive and rapid release of various pro-inflammatory molecules, including but not limited to INF-γ, TNF-α, IL-1, and IL-6. Macrophages, neutrophils, NK cells, and T cells are most often implicated in the pathogenesis of cytokine storm. The activation of primary T cells or immune cells’ lysis initiates the production of IFN-γ and TNF-α, which stimulate macrophages, dendritic cells, other immune cells, and endothelial cells to release more pro-inflammatory cytokines (Figure 4). The production of IL-6 is essential for cytokine storm because IL-6 activates T cells and other immune cells, thereby creating a positive feedback loop. The trigger activating CRS can be traditional therapeutic proteins and nucleic acids as well as small molecular drug allergens, whereas nanocarriers can amplify their toxicity. The analysis comparing the ability of adenoviral vectors and lipid-based carriers to induce cytokine production showed that lipid-based carriers exhibit higher immunostimulatory potential than viral vectors. The clinical translation of numerous nanoformulations designed for nucleic acid delivery was terminated in part due to the immune-mediated adverse effects [46].

## 7. Conclusions

It is evident that the programmability, biological compatibility, and modularity of nucleic acids assembled into multifunctional NANPs promotes this class of biologically active molecules into an innovative class of personalized therapeutics. To successfully translate these materials to the clinic, one has to recognize the importance of the indication, route of administration, and complexation of NANPs with delivery carriers. If delivered with a carrier via intravenous administration, the induction of cytokines and/or interferons by NANPs may lead to undesirable inflammation. Moreover, some carriers such as liposomes may also trigger CARPA upon systemic administration. However, the same type of cytokine or interferon response and complement activation by the carrier upon local administration may contribute to vaccine efficacy and improve the efficacy of immunotherapy. Experimental data from our laboratory provide several ways for controlling NANPs’ immunostimulatory properties. Among them are NANPs’ physicochemical properties (e.g., size, shape, sequence, connectivity), complexation with a delivery agent (e.g., lipofectamine, dendrimers), and route of administration (e.g., i.c., vs. s.c. or i.d.). Since the relationship between NANPs’ physicochemical/bioactive parameters and the immune system has just emerged, it is necessary to improve the current understanding of NANPs’ immunostimulatory properties for their successful translation to the clinic. We believe that the recent onset of mRNA vaccines to fight the COVID-19 pandemic will boost the field of therapeutic nucleic acids, including NANPs.
